# Integrity – a Driver of Integrated Care? Exploring Patient Experience in a Rapid Access, Short-Term, Outpatient Care Programme for Chronic Disease

**DOI:** 10.5334/ijic.10199

**Published:** 2026-11-02

**Authors:** Ashleigh Earnshaw, Amina Tariq, Mei-Ching Freeman, Hannah E. Carter, Kelly McGowan, Steven M. McPhail, Shannon Wallis, Sundresan Naicker

**Affiliations:** 1 Australian Centre for Health Services Innovation. School of Public Health and Social Work, QUT Centre for Healthcare Transformation, Faculty of Health, Queensland, Australia; 2 Darling Downs and West Moreton Primary Health Network, Springfield, Queensland, Australia; 3 West Moreton Hospital and Health Service, Ipswich, Queensland, Australia; 4 Metro South Hospital and Health Service, Digital Heath and Informatics Directorate, Woolloongabba, Queensland, Australia

**Keywords:** outpatient, patient experience, implementation science, qualitative evaluation, chronic care, integrated care

## Abstract

**Introduction:**

Globally, populations have been living longer with complex burdens of disease placing pressures on acute health systems and leading to the emergence of alternative models of care. There is a paucity of research exploring how patients experience these services. This study aims to explore patient experiences of a short-term, rapid access, outpatient service for chronic disease, with a focus on remote patient monitoring.

**Methods:**

The Preventative Integrated Care Service provides 16-days of intensive multidisciplinary support to patients with diabetes, chronic cardiac conditions and/or chronic respiratory conditions, located in Queensland, Australia. This qualitative study employed semi-structured interviews, with an interpretive description approach applied to analysis.

**Results:**

Thirty-three patients were interviewed. Patients reported overwhelmingly positive experiences, attributable to staff demonstrating integrity to a person-first approach to service delivery and care that felt appropriate and accessible.

**Discussion:**

The perceived accessibility and appropriateness of the service, and its contribution to the patients’ experience of care may suggest critical enablers to the service design in reducing the burden of treatment in this setting. The homogeneity of exceptional experience may offer further insights into enhancements in quality care for this patient cohort through an exemplar of positive deviance.

## Background

Services that provide rapid access to specialised care for people with chronic conditions have been proliferating, in response to overburdened health systems with long wait times. These services aim to support people at greater risk of poor health outcomes and reduce avoidable hospitalisations. Such models include rapid access clinics [1,2,3,4,5] and intensive outpatient care programs [6,7]. These models provide timely access to specialised care, facilitating either delivery of care in the outpatient setting and/or minimising use of hospital resources [3,4,6]. Key attributes include a person-centred approach, multidisciplinary or interdisciplinary team-based care, delivered through dedicated clinics that address specific specialty’s or conditions [5,6,8]. These services shift care from hospital to outpatient settings, which can flag concerns around care coordination, integration, and quality [9]. For patients with chronic diseases, these service models depend on the effective engagement of both patients who are required to be more active participants in their care (for example, the increased obligation on the patient to monitor and manage their symptoms), and service providers [6,10,11]. This is a complex process, requiring consideration of factors such as patient activation and empowerment [12], the burden of treatment and a patients’ capacity to enact the work required to manage their condition, and in turn, how care is tailored [11,13]. This includes considerations for multidisciplinary and person-centred care approaches [14,15] that are adaptable [10], and digitalisation of healthcare, particularly the use of remote patient monitoring technology [16,17,18]. However, person-centred care is difficult to define [14,15], often disaggregated into a series of actions required of clinicians [19], with comparatively less emphasis placed on the patients’ experiential perspectives.

Improving the patient experience of healthcare has gained momentum internationally [20,21,22] and is considered a cornerstone of healthcare quality [23,24,25]. Patient experience describes the patients’ perspectives of what and how healthcare services are delivered [26]. The use of patient reported experience measures (PREMs), which seek to quantify the patient experience often through survey measures, have proliferated in recent years. While there is utility in the application of PREMs [27,28,29], there is also imperative to deeply understand the human experiences of receiving healthcare [30]. This requires exploratory and applied methods that can disentangle complex experiential inquiries and construct practical and pragmatic outcomes, such as what matters most to patients in how care is delivered, and how this can be leveraged to improve quality. One method to achieve this is through the use of interpretive description [31]. Interpretive description is an adaptive and robust method for investigating complex experiences, arising from the interaction of multiple processes and factors. Consequently, it is used to understand people’s experiences in a practical way, with the goal of informing care and improving services. It is well suited to exploring complex healthcare experiences shaped by multiple interacting factors [31,32].

Much of the literature to date has focused on the effectiveness of patient- and service-level outcomes (such as hospital admission rates and length of stay), and perspectives of clinicians and administrators [1,3,5,6,7,10,33]. However, there is a paucity in the deeper examination of patient experiences of care within this unique clinical setting. This study aims to identify and explore factors related to the care experience for high-acuity patients within an integrated, chronic conditions rapid access clinic. This may inform future local service improvements, and wider scale and spread of similar models. A qualitative, cross-sectional patient cohort accessing care within a rapid access, intensive outpatient service in South-East Queensland, Australia will be used to explore the following key research questions:

1. What were patients experiences of receiving care in this service?
2. What were patients perceptions or experiences of using remote patient monitoring technologies to support their care in the outpatient setting?

## Methods

### Setting

The Preventative Integrated Care Service (PICS) operates across a hospital district located to the west of Brisbane, Australia. It operates one major acute teaching hospital (230 beds), four rural hospitals (approximately 20 beds each), a minor injury and illness clinic, and several community-based health services. Over 50% of residents in this area were shown to be living in areas of higher relative socioeconomic disadvantage, and experience higher rates of chronic disease and disability [34].

The PICS operates a 16-day rapid-access service for people with chronic cardiac conditions, chronic respiratory conditions, and/or diabetes delivered in the outpatient setting. The three clinical streams (cardiac, respiratory, and diabetes) of the service were sequentially established from November 2022, with the full complement of services operationalised from January 2023. See Supplementary File 1 for further information.

### Study design

This was a cross-sectional qualitative study. Interpretive Description was selected as it supports in-depth exploration of complex healthcare experiences, prioritises participants’ lived and subjective experiences as central to understanding, and generates practical insights to inform service improvement. This approach attenuates to lived experience, specifically, for future refinement of the health service or program [35]. See Supplementary file 2 for Positionality Statements. The Consolidated Criteria for Reporting Qualitative Research (COREQ) checklist [35] was used to guide both the study design and reporting of findings (Supplementary file 3). However, of note is the variation in qualitative methods and methodologies that means not all criteria may apply [36]. Persons with lived experience were not involved in the research design, due to financial constraints that prohibited remuneration of consumers, an expected practice [37,38], in place at the time of conceptualisation of the research.

### Recruitment and sample

Study eligibility required participants to be aged 18 years or older, have received care from the PICS (thereby meeting service eligibility), and have been discharged within two weeks from the service. Processes were in place to ensure those requiring (language other than English or Auslan) interpreter services and/or cultural liaison were able to participate in an interview, and were welcome to include a carer or support person. Ineligibility criteria were people who had not received care from the PICS (including carers or support persons individually), and patients who had concluded their care more than two weeks from provision of consent. This timeframe was congruent with the service parameters (i.e., 16-day model). We recognise that selection bias may be present in this study, as access to the service depended on referrer awareness. This may have limited access for some eligible individuals due to factors such as communication barriers, geographic location, and limited awareness of the service among healthcare providers. Interviews were conducted via telephone calls made from the researcher to the participant, to reduce participants’ financial and time costs, and minimise technological barriers to participation.

Recruitment was promoted verbally by PICS staff, via text message to patients (accompanying existing issuing of PREMs), and through flyers and posters located in the clinic. The research team had no prior contact with prospective participants. Recruitment occurred over four months (October 2024 to January 2025). Purposeful sampling methods were employed to ensure representativeness across demographic characteristics, where we estimated a sample size of 30–40 participants. This sample size was selected to promote representativeness in demographic and clinical participant characteristics and perspectives. Interpretive description emphasises insights through rich descriptions, continuous comparative analysis and development of robust interpretations, rather than an attempt at data saturation [32]. Consequently, sampling was continued until richness and depth in understanding of perspectives was agreed throughout the analytic process [32,39,40]. Participants received an AUD$60 grocery gift voucher as reimbursement for their time, following completion of the interview.

### Ethics

This study was approved by the West Moreton Health Human Research Ethics Committee (HREC/2023/QWMS/99335), and registered with the Australia and New Zealand Clinical Trials Registry (ACTRN12624000757516). All participants provided verbal and written informed consent prior to participation. Participation was voluntary, and participants were free to withdraw during the study period.

### Study materials and data collection

Semi-structured interview guides were developed that focussed on identifying the experiences of care in the PICS and perspectives or experiences in using remote patient monitoring through open-ended questions. These questions were refined through pilot testing with the research team and reviewed to enhance the conversational flow of the interview guide [41]. Data collection, analysis, and reflexive practise were undertaken concurrently by the research team. This enabled monitoring of data richness and iteration of the semi-structured interview guide (Supplementary file 4) accordingly. Field notes were taken before, during, and after interviews by a research team member (AE).

Interviews were 25–45 minutes in duration, and were conducted by telephone by a research team member (AE) and audio-recorded verbatim. Audio files were imported to, and transcribed in, Microsoft Word [42], and validated for accuracy and participant anonymity by AE. Reflexive journalling was undertaken within 24 hours of each interview. This documented reflections on the delivery and sequencing of interview questions, initial interpretations of participants’ key comments, and consideration of how the researcher’s clinical and professional background may have shaped interactions and interpretations. Reflexive practice also included ongoing memoing during data collection and analysis, documenting emerging ideas, patterns, and analytic decisions. In addition, the research team engaged in critical reflection on underlying assumptions, perspectives, and potential biases, including how these may influence data interpretation, to support transparency and rigour throughout the analytic process.

### Data analysis

Two researchers (AE and SN) familiarised themselves with the data by closely reading a subsample of transcripts (n = 8), listening to the audio recordings, and reviewing memos. They then came together and shared initial perspectives and engaged in discussion. This was then followed by predominantly inductive approach with coding undertaken by AE in NVivo 14.23.2, and collaboratively reevaluated for richness by SN with both researchers engaging in ongoing comparative analysis and iteration of codes (Figure 1). This approach was used to construct meaning from participant perspectives [32]. This also included ongoing reflexivity through memos and insights discussed between the two researchers (AE and SN). These insights were brought to the larger research team on a monthly basis for wider consensus. This process led to the construction of themes and subthemes.

**Figure 1:**
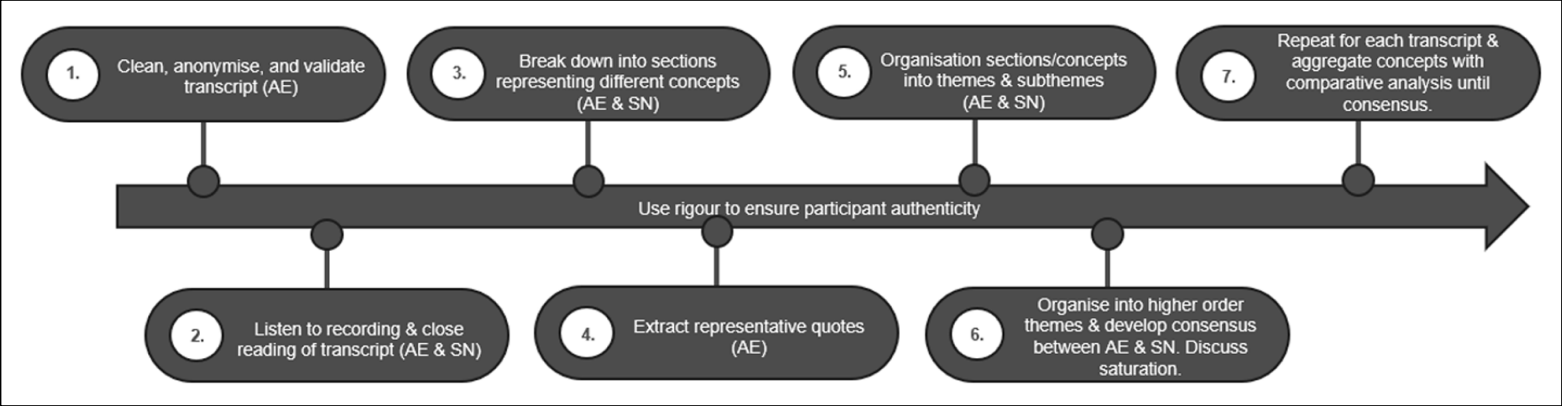
Qualitative analysis process.

### Rigour and reflexivity

Interpretive description has its epistemological grounding in constructivism [32,41]. As such, ongoing reflexivity was necessarily practiced throughout the study period through journaling, Memoing, and discussions with the research team to unpack positionality. Trustworthiness was addressed through ensuring representative credibility, constant comparison, analytical logic, and coherence [32]. Representative credibility was established through the use of two researchers undertaking analysis over multiple sessions, and sense checking additional data sources (PREMs (Supplementary file 5) and field observations) to develop deeper insight into the patient experience. Each subsequent transcript and audio recording analysed by AE and SN was compared to previous transcripts, and themes and subthemes adjusted accordingly to promote coherence. Analytic logic and coherence were ensured through maintenance of a clear audit trail, which was documented in databases and document libraries. This included photographic records of collaborative analysis, which demonstrate the development of a coherent interpretation of inductive findings [32]. Auditability was ensured through clear documentation, including a participant database, record keeping (digital, handwritten, and photographs of collaborative qualitative analyses results), and use of qualitative analysis software.

Personal biases and preconceived assumptions were discussed and challenged. This involved documenting the researcher’s initial expectations regarding data collection, acknowledging prior experience working with a similar patient cohort in clinical settings, and critically evaluating assumptions during weekly supervision. Strategies such as using open-ended questions, avoiding leading questions, and adopting a non-judgmental approach [32,41] were employed to facilitate discussions on participant perspectives.

## Results

Forty-seven service users indicated their interest in participating in this research. All were attempted to be contacted, resulting in thirty-three completed interviews (one per participant). Table 1 demonstrates participant demographic characteristics. There was a relatively even distribution of male (48%, n = 16) and female (52%, n = 17) participants. Half (n = 17, 51.5%) of the participants were aged 65 years or above, and two participants (6.1%) identified as First Nations. The highest proportion of participants had contact with the Respiratory specialty (39.4%, n = 13). The majority of participants were living in geographic areas classified as Major Cities (84.9%, n = 28) [43] and areas with higher socioeconomic disadvantage (as indicated by the Socio-Economic Indexes for Areas [44] decile one or two, 51.5%, n = 17). Target demographic characteristics (Supplementary file 6) were monitored throughout recruitment for representativeness. Detailed demographic data is available for all participants (Supplementary file 7).

**Table 1:** Participant characteristics.


DEMOGRAPHIC CHARACTERISTIC	NUMBER (%) n = 33

Sex	

Male	16 (48.0%)

Female	17 (52.0%)

Age group	

18-29 years	4 (12.1%)

30-49 years	3 (9.1%)

50-64 years	9 (27.3%)

65+ years	17 (51.5%)

Specialty	

Respiratory	13 (39.4%)

Cardiac	8 (24.2%)

Diabetes	6 (18.2%)

Multi-stream	6 (18.2%)

First Nations Status	

Aboriginal and/or Torres Strait Islander	2 (6.1%)

Not Aboriginal or Torres Strait Islander	31 (93.9%)

Socio-Economic Indexes for Areas decile	

1 and 2	17 (51.5%)

3 and 4	11 (33.3%)

5 and 6	2 (6.1%)

7 and 8	3 (9.1%)

9 and 10	0 (0.0%)

Remoteness classification ^a^	

Major cities	28 (84.9%)

Inner regional	5 (15.2%)


*Notes*: ^a^ Accessibility/Remoteness Index of Australia Plus (ARIA+).

Three themes, each with two subthemes, were identified. Firstly, integrity to a “patient-first” approach described how patients experienced care, constituted of two subthemes – authenticity in the therapeutic alliance, and responsive and person-centred actions. The second theme, appropriateness and accessibility of care, described the “what” of care delivery, reflected in the two subthemes – personalised and knowledgeable support, and accessible and timely care. The combination of these two themes produced an exceptional patient experience, where the patient felt that they were being treated as a whole person. Finally, acceptability and normalisation of Remote Patient Monitoring was described, which suggested that patients were comfortable with using the technology (subtheme), and that it was perceived to provide a sense of safety during treatment (subtheme). The relationship between themes is summarised in Figure 2.

**Figure 2:**
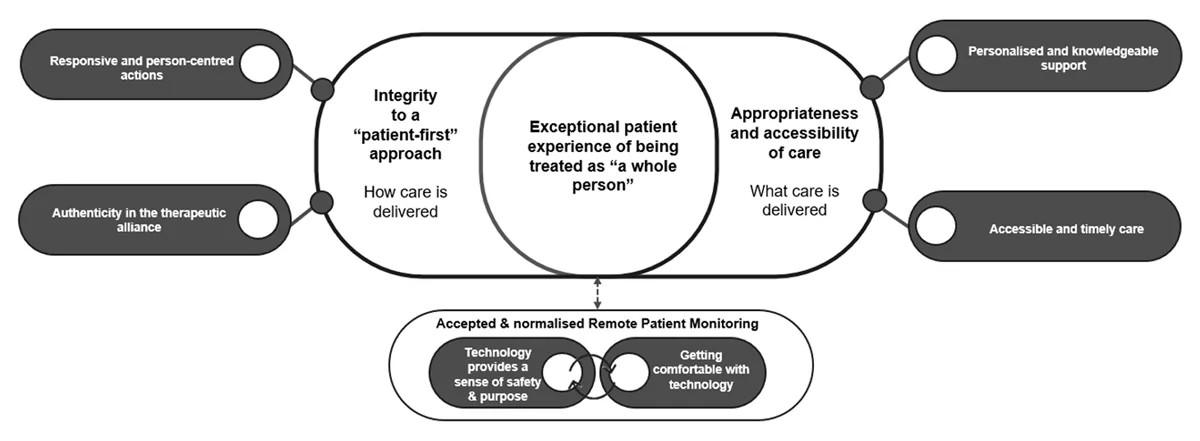
Relationships between themes and subthemes.

### Theme 1: Integrity through a “patient-first” approach – Aligning Values, Intentions, and Actions

Across the participant accounts, a strong throughline emerged: participants perceived that staff at PICS consistently aligned their values, intentions, interpersonal relationships, and actions in a way that honoured the principle of “putting the patient first.” This integrity was not only felt in one-on-one interactions, but also in the coordination between clinicians, support staff, and the wider health system. This shaped how care was experienced as compassionate, trustworthy, respectful, and tailored; and underpinned the high quality of all aspects of service delivery.

*“If they say they’re gonna do something, they do it…they don’t think about it for six months and then do something”. C09, Respiratory stream*.

Participants described that through strong relationships their concerns were heard and acted upon, and as such, they felt as though were treated as a “whole person”, as opposed to a disease or number moving through the health system. This was perceived as a surprise to some patients, who described the service as “life-changing” and “nothing I’d received before”.

*“They did (take time with me) and I felt as if they cared… sometimes…your doctor is too busy clicking away on his computer, but…the people in there (PICS) were very good… I’m gobsmacked at…the…care I’ve been given. I never thought I’d come out of the hospital and…have this extent of people and in my corner helping me out and guiding me on the right way”. C20, Cardiology stream*.

#### Subtheme 1: Authenticity in the Therapeutic Alliance

Throughout all interviews, participants described a sense of feeling respected, trusted, and heard in their interactions, which imparted a sense that the staff members were compassionate – genuinely wanting to help the participant resolve their issues and “get their health back on track”. Participants experienced this as the staff taking extra time prior to the participants’ appointment and with the participant to understand their medical history and social circumstances, and actually “getting to the bottom” of long-standing issues or concerns.

*“They treat you beautifully. I think they’re very interested in you and how you’re living your life…I really can’t explain… I have never come across it before… not ever. Because you go to a doctor, you go to hospital like shoot you in and shoot you out – “Bye-bye, go home, goodbye…. We’ll give you some antibiotics, we’ll give you some steroids. That’s it”. We won’t get to the root of the problem… That isn’t what happened here (at PICS)”. C34, Respiratory stream*.

Simultaneously, participants described being met with an openness and environment that was free from judgement about social circumstances and past experiences. This in turn enhanced their willingness to engage with the clinicians and care more broadly.

*“I sorta felt I could talk to them about anything…I’ve been to… certain places before and told them a bit about my life…it almost…a bit of like sense of judgement, when it’s like at PICS, I was telling him everything I used to do before I had diabetes and you know just anything. And…there’s no judgement. Felt very welcomed and they were very helpful”. C07, Diabetes stream*.

#### Subtheme 2: Responsive and person-centred actions

Participants described that actions taken by the PICS staff were reflective of their desire to ensure the patients’ needs were met in a timely manner, which positively reinforced the therapeutic alliance and positive experiences with the service. These actions were not only related to direct healthcare provision and participation, but also in providing emotional support to the patients’ support network.

Overwhelmingly, participants felt that their needs were placed foremost in their care. Despite many participants reporting seeing several members of the multidisciplinary team, they felt that each subsequent clinician was aware of their healthcare needs and broader personal circumstances. This meant that participants felt that they did not have to repeatedly “re-tell their treatment journey”. Participants also positively remarked that they were able to see multiple clinicians within one visit with ease and limited waiting times, which “made the most” of their time while unwell.

*“And they worked together… they worked as a team. It wasn’t like you saw this person and then this person. The team communicated about, you felt as though you were their main focus… I’d see one person, and then instead of … just sit here and wait, that person would actually go and talk to my next appointment…Say I was seeing the nurse then say to the dietitian “I’m finished with <<name>>, this is what we discussed”…It just felt like they were in-sync”. C43, Diabetes stream*.

Participants reflected on the tangible actions they were aware had been taken after their appointments (e.g., communicating with hospital staff, actioning pathology, or medication pack requests) which positively reinforced the authentic therapeutic alliance. This sense of authenticity also fostered engagement and inclusion of the participants in their own self-management and decision-making. Participants explained that by feeling respected and heard, they had a strong voice in their own decisions and preferences for care, as exemplified by the following detailed account.

*“Well, my (emergency department) presentations in general have decreased. (Doctor) is taking more of a hands on to tweaking medications… working with my GP as well, working with other specialists… Immunology and with…another specialist… And when I’ve had issues with the health system since she’s gone out of her way to help. I had some issues with the local hospital and she worked with the doctors at the local hospital…to get management plans in place to prevent the issues from continuing to happen”. C42, Respiratory stream*

Participants communicated a sense of feeling emotionally supported in times of need. This was evident through actions taken that demonstrated this emotional support, such as “going above and beyond” providing exceptional healthcare within the constraints of the service. Participants were surprised by, and deeply valued, this level of emotional support.

*“They made me feel as though I wasn’t just another number, you know, like when you’re going to Casualty (emergency department), I just feel terrible because you’re just another … Whereas the PICS team, they’re just so good. They’re on the ball straight away. And not only that, the emotional support they give you is fantastic. The support they gave my husband was really, really fabulous because he was so upset about me and they just talked to him while I was waiting for the ambulance”. C09, Respiratory stream*

### Theme 2: Appropriateness and Accessibility of Care – Participating in care that felt appropriate to my needs

Appropriateness refers to the relevance of the innovation of intervention as perceived by those receiving it; distinct yet similar to acceptability, which refers to the perceived palatability of the intervention [45]. Participants consistently described feeling that they were able to access care that met their medical and psychosocial needs, within a timely manner. This was evident in two major dimensions, reflected as subthemes – how care was tailored and adapted to individual needs (personalised and knowledgeable support), and multidimensional aspects of accessibility such as multimodal service delivery and rapid access to care (accessible and timely care).

#### Subtheme 1: Personalised and Knowledgeable Support

Throughout participant accounts of the care they participated in, a strong sense of receiving care that was highly specialised and tailored to the patient’s medical and psychosocial needs was described. To participants, this was experienced as participating in conversations that allowed complicated health information to be explained to them clearly and without jargon.

*“(The staff) explained everything so that I could understand, didn’t just tell me what to do, they explained carefully so that I understood what was going on. And I sometimes I don’t understand things very well (due to a brain injury) … And they took their time as they needed it to and make sure I understood”. C13, Cardiology stream*.

As noted in Theme 1, participants often described a sense of being deeply listened to and taken seriously, and in turn, they received advice that was tailored to their unique healthcare needs and living situations. This advice extended beyond basic education, to new practical assistance, that participants felt were derived from many years of clinical experience of the staff. This personalisation was experienced as exceeding most other care provided for their condition, compared to the participants’ past experiences.

*“So you felt like they were really, really in tune with what it’s like to have diabetes and took into consideration all of your personal factors, not just almost like an algorithm…not only did they deal with the diabetes, it…wasn’t only focussed on my health issue…it was exceptional”. C43, Diabetes stream*.

In turn, participants felt their knowledge and confidence in self-managing their condition and accessing emergency care when clinically appropriate had improved as a result of their engagement with the PICS.

*“There are a couple of times where if I didn’t have all the knowledge they gave me, and the help they gave me, you probably would have re-presented at the hospital, because you didn’t know where to turn”. C27, Diabetes stream*.

#### Subtheme 2: Accessible and Timely Care

Participants described several attributes of accessible care when discussing their experiences with PICS. These included physical accessibility of the clinic infrastructure, flexibility in how patients could participate in their care, and timeliness of care. Participants stated that the proximity of the clinic to the hospital was positive, both in terms of having these inpatient services close by if needed, and because of the transport infrastructure (rail and bus networks), that service the area. Further, the free, onsite parking, and disability-friendly accommodations (e.g., wheelchair ramp, wide corridors, and large clinic spaces) were contrasted by participants to attending other health services where such considerations are absent.

*“Well, it’s…really set up for handicapped people…The handicapped spots are right beside the door and there’s plenty of space to negotiate, push a wheelchair…I find the hospital, our hospital is so old. The corridors are quite narrow and some of the rooms are almost impossible to get into”. C09, Respiratory stream*.

For participants who had mobility issues, poor functional abilities, or experienced social isolation, the offering of home visits greatly enhanced the participants’ perceived ability to engage in care, and reinforced perspectives that the clinicians were taking actions to ensure their health and social needs were met.

*“I definitely needed them to come into the home because if I’d had to get myself into the car and…find a park and then find my way into…wherever I had to go to interact with these people, it would have just worn me out”. C27, Cardiology stream*

Participants who experienced repeated or worsening illness often described long delays in other parts of the health system, delays they felt were unsafe or anxiety-inducing. In contrast, the PICS responded quickly, usually within a day of referral or contact. This rapid access to clinicians and specialised care was described as reassuring and lifesaving, particularly by those who felt unstable, overwhelmed, or unsure where else to turn.

*“Getting in to him (private specialist)…or calling him about anything. It was like a month wait… And given how sick I’d been, it just wasn’t really… the safest… option for me at that time. Like I was so fragile”. C19, Respiratory stream*

### Theme 3 – “Someone’s Looking Out for Me”: How Participants Accepted and Normalised Remote Monitoring

#### Subtheme 1 – “It’s no big deal”: Getting Comfortable with Technology

Despite initial concerns about their own technical ability, most participants, particularly those under 65 years, described the setup and use of remote monitoring devices as straightforward. Some reported needing help from family members or clinicians to get started, while others found the process intuitive with minimal need for training.

*“There was supposed to be a training thing. They called me to do it and I went “You know, you sent me the links like I’ve just looked, it was very simple and I’ve already set it up”. So they did offer the training. It was just, very… self-explanatory setup”. C42, Respiratory stream*

Even participants who described themselves as “not tech savvy” expressed openness and willingness to try, often surprising themselves with how quickly they adapted.

*“I have a problem with using technology. My only concern would be that I would muck it up. I’m happy to give it a try…My problem would be that I would muck it up and I might have to ring people saying “what the hell am I doing here?”. C34, Respiratory stream*

Participants who used intermittent monitoring described that embedding its use into their daily routine (such as undertaking readings after waking) provided a seamless management strategy reducing initial reservations about its use. These reservations were less pronounced in patients using continuous monitoring, who often reported that they forgot they were wearing the device, and found it easy to adapt to changing the device on a regular basis.

*“Well this I do first thing in the morning, so…it fits in. It’s…very easy. I’ll make myself a herbal tea and then I’ll sit down… I wait 5 minutes and then I take my blood pressure and then I do the oximeter and yeah…it’s 2 minutes and it’s done”. C20, Cardiology stream*.

#### Subtheme 2 – A Sense of Safety and Purpose: “A second pair of eyes”

Participants described Remote Patient Monitoring as more than a technical tool, it gave them a feeling of being watched over, even from a distance. Many were surprised that clinicians were actively monitoring the data and responding in real time. This sense of surveillance-with-care helped participants feel safer, especially when their health was unstable. What began as scepticism or indifference for some turned into deep appreciation after clinicians intervened based on their readings.

*“At first I was like, oh, this is really annoying…But I think like after them helping me through that moment (acute deterioration), I was like, it’s actually really good for them to know how I’m doing from a distance without having to call me every day”. C19, Respiratory stream*

## Discussion

A strong theme emerged from this study, whereby participants felt that the staffs’ values, actions, and care all demonstrated that they were “putting me first”. This was demonstrated in two ways – firstly, how care was delivered (integrity to a “patient-first” approach), and secondly, what care was delivered (care that felt appropriate and accessible). While these findings align with the conceptualisation of the patient experience offered by Bull, Byrnes [26], and the nexus of compassion and clinical competency offered by Sharp, McAllister [46] in exceptional acute care, we proposed that integrity to a “patient first approach” was a unique product of consistent alignment of values and actions, which had a profoundly positive impact on the patient experience of care.

Integrity has been defined as a strong adherence to a moral principle [47]. In this research, we have defined integrity as it applies to the moral principle of “putting the patient first”. This reflects a sense of both personal integrity, and the notion that integrity may act as a social construct embedded in a context that upholds specific behaviours as important, as described by Calhoun [48] and Tyreman [49]. Importantly, the integrity to a person-first approach represents a deeper relational connectedness that may facilitate higher quality care more generally [50].

A key aspect underlying relational connectedness is trust. For participants this meant being trusted by, and having trust in, their health professional; and feeling an absence of judgment in their circumstances. Much research within the healthcare setting has emphasised the importance of compassion – a complex and multifaceted value that is typified by a desire to help those in need through human connection and understanding [51]. In the inpatient setting, the combination of compassion and clinical competence has been demonstrated to facilitate patient-centred care [46], for which our findings are consistent. And while clinicians’ personal and professional values drive compassionate healthcare delivery [51], we argue that in this rapid-access, short-term care setting, compassion is necessary, but not sufficient to achieve the exceptionally positive experience of care noted within this service. Integrity to the person-first approach goes beyond providing compassionate care to patients and their loved ones – it is the repeated alignment between intentions and actions with, and on behalf, of the patient [52].

PICS delivers a multimodal model of care that is tailored to patient needs. This meant that the threshold for patient activation is typically lower than for standard health services, consequently reducing barriers to participation [53,54]. These considerations reflect a conscious effort in PICS service design and delivery to actively address sociodemographic considerations that impact the local population (for example, higher proportions of the population experiencing socioeconomic disadvantage and disability). These considerations align with principles of burden and capacity in Burden of Treatment Theory. Burden of Treatment Theory posits that factors such as health literacy, disability, and transport can reduce a person’s (and their caregiver’s) capacity to engage in healthcare services and the work (burden) required to manage their condition [11,13]. Patient’s positive views on multimodal service delivery and accessible infrastructure reflect the value of reducing care burden through addressing issues surrounding transport and access. This has been demonstrated to be a concern in the Australian healthcare setting for people with multimorbidity [55] and may require further exploration in alignment with the principles of Universal Design in future research, which incorporates the foundational principles of accessibility and equity when developing complex interventions like new models of care [56]. Further, the rapid access nature of the service and high degree of communication between staff [55,57] may reflect service delivery elements that reduce the need for patients’ (and caregivers’) to mobilise resources to engage with the health system, freeing capacity to undertake the workload of treatment and monitoring.

Additionally this study sought to explore patients’ perceptions and experiences in using Remote Patient Monitoring. The use of Remote Patient Monitoring was found to mutually reinforce the therapeutic relationship and support chronic disease self-management. The majority of patients’ perceptions and attitudes towards Remote Patient Monitoring indicated a high degree of normalisation and acceptance of the use of technology to support care. This is reflective of the ubiquitous nature of technology in today’s society, and potential expectation that it will be used in care delivery. While patients expect digital health technology to be used, they strongly prefer its application to be purposeful – specifically, with health professionals actively monitoring data. This ensures the data generates actionable insights, enabling care teams to intervene proactively when signs of deterioration appear or further refine a patient’s treatment plan. This feedback loop to patients and sense of safety may have enhanced the perceived value of Remote Patient Monitoring, reinforced the acceptance of the technology, strengthened the therapeutic alliance, and improved patient knowledge and self-management of their condition. These findings are congruent with other studies examining Remote Patient Monitoring in patients with chronic disease [58,59,60].

Where patients did not perceive themselves as technologically savvy, many expressed they were willing to try Remote Patient Monitoring, particularly if they were taken through an onboarding process. This highlights the widespread acceptance and normalisation of technology use, while emphasising the importance of human involvement in care. Two patients (both aged 65+ years) expressed resistance to Remote Patient Monitoring, citing personal preferences for written self-monitoring. While previous research suggests that older patients are more likely to demonstrate resistance to digital health technologies [61], the majority of participants willing to try the technology suggests a shift towards expectations of technology inclusion in healthcare. Throughout discussions about use of technology, the importance of human connection (and hence, adequate resourcing) was emphasised – through onboarding processes, use of data, and responsive actions. This reinforces the notion that the social interaction between patient and clinician surrounding the technology is a complex interplay that can enhance its acceptability and usability [62].

### Strengths and Limitations

This study provides a robust qualitative examination of a novel, short-term, rapid access, outpatient service operating within Queensland, Australia. This study was restricted to a single site, due to the novel nature of the service. As such, findings may not be generalisable beyond this service model or to health systems serving populations with notable differences in the socio-demographic profile. Further, we acknowledge the limitations associated with involvement of consumers in the conceptualisation of this research. Future research may wish to test the relationship between patients’ experiences of care and their health outcomes, to ascertain whether the foundational components of how (integrity to a person-first approach) and what (appropriateness and accessibility of care) care was delivered is associated with, or predictive of, patient activation and chronic disease self-management.

## Conclusions

This research provides novel insight into patients’ experiences of a digitally enabled, rapid access, short-term service for chronic disease management in South-East Queensland, Australia. Participants described their experience of care as profoundly positive, an outcome shaped not simply by the delivery of appropriate clinical services, but by the moral and relational integrity with which those services were enacted. This was evident in the way staff consistently prioritised the patient as a whole person, through authentic, respectful relationships; seamless team communication; and a willingness to act on patients’ needs in timely and meaningful ways. These findings reinforce the importance of aligning service design with deeply held values and suggest that integrity may serve as a foundation and a catalyst for patient-centred excellence. Remote patient monitoring was experienced as a valuable adjunct to care, enhancing trust in clinicians and patients’ capacity to self-manage their health. Its perceived value depended not only on functionality, but on meaningful clinician engagement with the data it generated. To sustain and expand its benefit, ongoing technical support and proactive intervention are recommended. These findings underscore the importance of fostering healthcare environments that support relational integrity, not only in principle but in practice. When services are built around trust, responsiveness, and authentic connection, patients notice, and their experiences reflect it.

## Additional Files

The additional files for this article can be found as follows:

10.5334/ijic.10199.s1 Supplementary File 1. Preventative Integrated Care Service workforce profile.

10.5334/ijic.10199.s2 Supplementary File 2. Positionality statements.

10.5334/ijic.10199.s3 Supplementary File 3. Consolidated criteria for reporting qualitative studies (COREQ): 32-item checklist.

10.5334/ijic.10199.s4 Supplementary File 4. Semi-structured interview guide.

10.5334/ijic.10199.s5 Supplementary File 5. PICS Consumer feedback survey.

10.5334/ijic.10199.s6 Supplementary File 6. Target participant sample demographic characteristics.

10.5334/ijic.10199.s7 Supplementary File 7. Detailed participant characteristics.
