# Supplementary material for: Integrity – a Driver of Integrated Care? Exploring Patient Experience in a Rapid Access, Short-Term, Outpatient Care Programme for Chronic Disease
Source: Int J Integr Care. 2026 Nov 2;26(4):6. doi: 10.5334/ijic.10199 (PMC13741939; doi:10.5334/ijic.10199)

Supplementary File 1. Preventative Integrated Care Service workforce profile

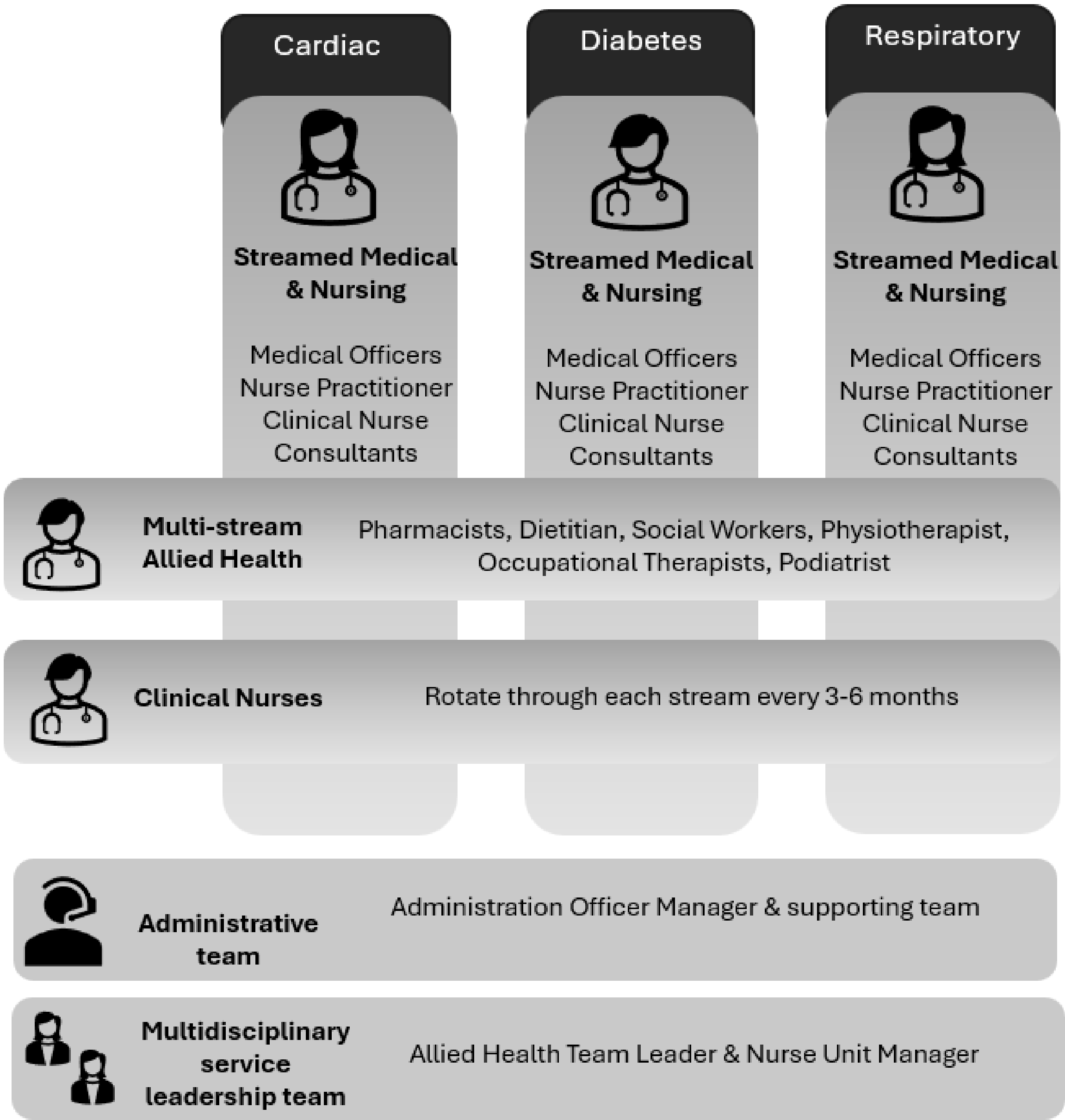

Supplement: Supplementary file 1 [file ijic-26-4-10199-s1.pdf]
