# Supplementary material for: Integrity – a Driver of Integrated Care? Exploring Patient Experience in a Rapid Access, Short-Term, Outpatient Care Programme for Chronic Disease
Source: Int J Integr Care. 2026 Nov 2;26(4):6. doi: 10.5334/ijic.10199 (PMC13741939; doi:10.5334/ijic.10199)
Supplement: Supplementary file 2 [file ijic-26-4-10199-s2.pdf]

Supplementary File 2: Positionality Statement

Ashleigh Earnshaw – BHlthSci (Nutrition & Dietetics), BPsycSc (Hons), MPH

I approached this study from an interpretivist standpoint, understanding lived experience as socially situated, subjectively meaningful, and irreducible to measurable outcomes. My goal was to construct understanding of participants' experiences from within their own frames of reference, while remaining transparent about the ways my own biography, professional training, and institutional location shaped that process. I bring nearly fifteen years of experience across the Australian healthcare sector, including clinical practice as a dietitian, project management, health service planning, and performance evaluation. This range of roles has accustomed me to thinking across individual, service, and system levels simultaneously, a disposition that was both useful and, at times, in tension with the experiential depth that qualitative inquiry demands.

As an Anglo-Saxon woman living with two chronic conditions, I held a partial experiential connection to the participant group. I could recognise, in their accounts, the texture of managing daily life while feeling unwell, the fatigue, the negotiation of symptoms, the ways illness reorganises ordinary routines. This partial identification created moments of interpretive attunement that I believe enriched my engagement with participants' stories. At the same time, I was acutely aware of the limits of this connection. My conditions differ from those addressed by the service, and I am not subject to the omnipresent threat of acute deterioration or hospitalisation that characterised participants' lives during the study period. I was therefore careful not to over-identify or to allow personal resonance to substitute for attentive listening. Where I noticed myself anticipating a participant's meaning rather than remaining open to it, I used this as a signal to slow down and probe further. The partial insider position I occupied as a person with chronic illness required ongoing reflexive management to ensure it remained a resource for understanding rather than a source of projection.

My clinical formation as a dietitian has profoundly shaped how I listen and how I make sense of what people tell me. That work was grounded in Motivational Interviewing and Acceptance and Commitment Therapy, approaches that trained me

to attend to ambivalence, self-efficacy, and the social and emotional dimensions of health behaviour, rather than defaulting to biomedical framings. This clinical orientation was a genuine asset in conducting interviews sensitively and in recognising the relational and emotional texture within participants' accounts. It also, however, carried risks I needed to actively manage: a tendency to hear experience through a behaviour-change lens, or to focus on what participants described doing rather than what their experiences meant to them. I surfaced and interrogated these tendencies through reflexive journalling and discussion with the research team throughout data collection and analysis.

A significant portion of my clinical career was conducted via telephone, delivering diet and lifestyle support to patients managing chronic conditions remotely. I entered this mode of interaction with established skills in building rapport, reading verbal cues, and managing the relational work of a conversation without visual reference. I had come to understand telephone-based encounters as capable of generating genuine connection and, in some respects, of moderating power dynamics, removing the pressure to physically present, and creating a degree of neutrality around appearance and setting. I remained reflective, however, about whether this comfort risked obscuring gaps in what telephone interviews can access, and whether there were dimensions of participants' embodied or environmental experience that a different mode of encounter might have reached more readily.

At the time of conducting this research I was employed by the health district within which the service under examination operates. I held no clinical role and had no direct professional involvement with the service itself, but I carried a detailed working knowledge of the organisational context, the local geography, and the population the district serves. When participants described navigating the hospital campus, managing transport to clinic, or making sense of service communications, these were environments and processes I knew from the inside. This insider familiarity offered genuine interpretive advantages because I could contextualise accounts with confidence and was unlikely to misread organisational references. It also, however, created an obligation to resist the normalisation of features of the service environment that participants might experience as confusing, burdensome, or alienating. I worked deliberately to hold multiple interpretive positions simultaneously ,as a person with chronic illness, as a clinician, and as an organisational insider,

interrogating how each framing opened and closed different meanings within the data.

Across the research process, I used reflexive practices to support transparency and rigour. I kept a reflexive journal from the start of data collection, where I noted my reactions, moments of discomfort or connection, and early ideas about what the data might mean. Writing analytic memos helped me track how my interpretations developed over time and made my thinking visible.

I also discussed the data regularly with the research team, particularly with SN. Working closely together allowed us to interpret meaning collaboratively, question each other's assumptions, and strengthen how we made sense of the findings. These conversations helped surface perspectives I might not have recognised on my own.

Dr. Sundresan Naicker

I am a mid-career researcher based at the Australian Centre for Health Services Innovation (AusHSI) at Queensland University of Technology, with training and practice spanning implementation science, digital health, and applied health services research. My academic formation has been rooted in the positivist tradition, completing a PhD in Medicine at the University of Sydney and undertaking postdoctoral work in large-scale randomised controlled trials and implementation studies. Over the past decade, my work in real world service improvement across hospital wards, outpatient clinics, community organisations, and policy settings has deepened my sensitivity to the lived, relational, and context specific dimensions of healthcare, which cannot be fully captured by metrics or system level outputs.

My professional familiarity with the health system as a researcher means I must be especially careful not to confuse systemic knowledge with an understanding of what it feels like to rely on it as a patient. This shift in orientation has required me to be deliberate and reflective about how my positioning as a health systems researcher and implementation scientist might shape what I attend to, what I assume, and what I risk missing. As a male researcher of South Asian and African heritage working within a predominantly Anglo-Australian institutional setting, I am aware that my cultural location and professional identity shape how I approach participants, interpret their accounts, and make sense of experiences that may differ substantially from my own. Study participants come from diverse social, economic, and cultural backgrounds and navigate a health system that may feel, at times, impersonal or difficult to access.

My prior work in implementation science has sharpened my awareness of a particular bias I carry: a tendency to view service encounters primarily through the lens of uptake, adherence, and system optimisation. I have had to actively interrogate this framing throughout the analytic process. Lived experience is not a proxy for implementation outcome; it is its own form of knowledge. Participants in this study are not merely recipients of a service model; they are people navigating illness, uncertainty, and care at a vulnerable point in their lives. Engaging qualitatively with their accounts has required me to slow down my interpretive instincts, resist the pull toward process-level explanations, and remain open to the emotional, relational, and deeply personal dimensions of what rapid outpatient sub-acute care means for those who receive it.

I have approached this study from a broadly interpretivist stance, informed by phenomenological sensibilities. My goal has been to understand experience as participants themselves make sense of it, rather than to map their accounts onto pre-existing theoretical or service improvement frameworks. To support this, I have engaged in ongoing reflexive practices throughout data collection and analysis: maintaining a reflective journal, engaging in peer debriefing with co-investigators, and using analytic memoing to surface and interrogate assumptions as they arose. Where I noticed my interpretive lens pulling toward service-system explanations, I returned to the data to ask what participants themselves were conveying about meaning, agency, safety, and connection.

Ultimately, I understand my positionality not as a source of contamination to be controlled for, but as a constitutive element of the interpretive process,one that must be named, interrogated, and held accountable throughout the research. My aim has been to bring the rigour and discipline of my research training into productive dialogue with a genuine openness to human experience, in ways that honour the trust participants have placed in sharing their stories with us.
