# Supplementary material for: Integrity – a Driver of Integrated Care? Exploring Patient Experience in a Rapid Access, Short-Term, Outpatient Care Programme for Chronic Disease
Source: Int J Integr Care. 2026 Nov 2;26(4):6. doi: 10.5334/ijic.10199 (PMC13741939; doi:10.5334/ijic.10199)
Supplement: Supplementary file 4 [file ijic-26-4-10199-s4.pdf]

Supplementary file 4: Semi-structured interview guide

This semi-structured interview guide is designed to elicit information for the purposes of objective two.

Note: This interview guide is semi-structured by design. These questions will be adapted to either the pre- or post-implementation phase of this study and will refer to the most contextually relevant solutions being used by the participant(s) at the time of this interview.

Introduction

Hi, my name is <name> from the Queensland University of Technology. Thank you for taking the time to participate in this research on the how you're finding using technologies to help monitor your health while on the Preventative Integrated Care Service.

If you give your permission, we will record this conversation to allow for analysis. The audio recording and any analysis will only be accessible to the QUT research team, and we will make sure that other people cannot identify your responses.

Please remember that you are free to decline to answer any question, request your response not be recorded, or withdraw at any time without providing a reason.

There are no right or wrong answers, we are interested in understanding your experiences and perceptions.

We really appreciate your time and willingness to speak with us. Do you have any questions before we get started?

Firstly, we'll talk a bit about you and your health. Then we'll move on to discussing your experiences with the service in more detail.

1. Would you please be able to tell me a little bit about yourself?

2. In general, would you say that your health is excellent, very good, good, fair, or poor?[1]

3. Which of the following health conditions has your doctor told you that you have (select all that apply):
   ☐ Type 1 Diabetes
   ☐ Type 2 Diabetes
   ☐ Heart failure
   ☐ Another heart condition
   ☐ COPD
   ☐ Another lung condition

4. How long have you been living with this condition/these conditions for?

[1] Australian Bureau of Statistics. (2022). *Patient Experience Sur vey (PEx) July 2021 to June 2022 Household Survey Questionnaire.* Module 1 Self-assessed health status (SAH). Question SAH_Q01

5. What other health services or practitioners do you usually see? (E.g., GP, psychologist, dentist)
    a. What do you see them for?
    b. Does anything stop you from seeing them? (E.g., cost, waiting time is too long, transport is an issue)

Now, let’s discuss your experience with PICS. We’ll start off with talking about PICS in general, then discuss the technology component of the service.

6. How did you find out about PICS?
7. What were your goals/what were you hoping to achieve by working with the PICS team? E.g., help control blood sugar levels, stay out of hospital.
8. How have you met with the PICS team members? E.g., in the clinic, over the phone, virtually?

Now, lets talk about the monitoring equipment and technology.

1. How “tech savvy” did you consider yourself before using this monitoring equipment?
   What technologies/devices do you already use in your daily life? For example, emails, social media apps, a smart phone.
2. Can you please talk me through how you were shown what to do to take your own measurements by the PICS team? Was there anybody else that was also shown how to use to it assist you? (For example, a spouse or child).
3. And at the start, what were your first thoughts about using this monitoring equipment to manage your <<condition>>?
4. And what were your thoughts and feelings about using this equipment as time progressed?
5. Did you have to learn any new skills to use the monitoring equipment?
6. At the start, how confident did you feel in using the equipment/technology to manage your <diabetes/respiratory condition/heart condition>? What about as time progressed?
7. How useful did you think the monitoring equipment would be in helping you to <<achieve your goal noted above>>?
8. Walk me through a day in the life of <<name>>. How does using the monitoring equipment fit within your usual daily routine?
    A) Were there any prompts or reminders you gave yourself to use the monitoring equipment?
    B) Did anybody else help you remember or support you to use the monitoring equipment?
    C) Is there anything that the monitoring equipment interferes with? (e.g., going swimming)
    D) How has your daily routine changed to accommodate the remote biometric monitoring? For example, do you have to do any pre-planning around your schedule, are there any out of pocket costs to you as a direct result from using it?
9. What are some of the challenges you’ve had with using remote monitoring technologies?
    a. Are there any aspects of remote monitoring technologies that you don’t like?
    b. Any that you do like?
