# Supplementary material for: Integrity – a Driver of Integrated Care? Exploring Patient Experience in a Rapid Access, Short-Term, Outpatient Care Programme for Chronic Disease
Source: Int J Integr Care. 2026 Nov 2;26(4):6. doi: 10.5334/ijic.10199 (PMC13741939; doi:10.5334/ijic.10199)
Supplement: Supplementary file 5 [file ijic-26-4-10199-s5.pdf]

Supplementary File 5 – PICS Consumer feedback survey

Consumer feedback survey question and responses	n (%)
How do you rate the care that you received?	
Very good	245 (92.1%)
Good	15 (5.6%)
Neutral	0 (0.0%)
Poor	6 (2.3%)
Very poor	0 (0.0%)
Do you feel the care you received was right for you?	
Strongly agree	217 (81.6%)
Agree	43 (16.2%)
Neither agree or disagree	0 (0.0%)
Disagree	0 (0.0%)
Strongly disagree	6 (2.3%)
After receiving care with PICS, do you feel more confident in taking care of your chronic condition?	
Strongly agree	132 (49.6%)
Agree	94 (35.3%)
Neither agree or disagree	40 (15.0%)
Disagree	0 (0.0%)
Strongly disagree	0 (0.0%)
Were you on Remote Patient Monitoring?	
Yes	79 (29.7%)
No	187 (70.3%)
If you were on Remote Patient Monitoring, do you feel it helped you stay out of hospital?	
Yes	59 (74.7%)
No	20 (25.3%)
If you had not received care through PICS, would you have gone to the Emergency Department or called an ambulance for your condition?	
Yes	134 (50.4%)
No	90 (33.8%)
Not applicable – I was not unwell enough to call an ambulance or go to the Emergency Department	42 (15.8%)
