# Supplementary material for: Integrity – a Driver of Integrated Care? Exploring Patient Experience in a Rapid Access, Short-Term, Outpatient Care Programme for Chronic Disease
Source: Int J Integr Care. 2026 Nov 2;26(4):6. doi: 10.5334/ijic.10199 (PMC13741939; doi:10.5334/ijic.10199)
Supplement: Supplementary file 6 [file ijic-26-4-10199-s6.pdf]

Supplementary file 6: Target participant sample demographic characteristics

Demographic characteristic	Target Proportion (%)
**Sex**	
Male	50.3%
Female	49.7%
**Age group**	
18-29 years	4.4%
30-49 years	16.1%
50-64 years	33.9%
65+ years	45.6%
**Clinical Stream**	
Respiratory	35.7%
Cardiac	21.6%
Diabetes	28.1%
Multi-stream	14.6%
**First Nations Status**	
Aboriginal, Torres Strait Islander, or Aboriginal and Torres Strait Islander	6.8%
Not Aboriginal or Torres Strait Islander	93.2%
**Socio-Economic Indexes for Areas (SEIFA)**	
Deciles 1 and 2	63.8%
Deciles 3 and 4	16.6%
Deciles 5 and 6	4.9%
Deciles 7 and 8	7.6%
Deciles 9 and 10	2.5%
Missing/not reported	0.5%
Outside of catchment	4.2%
**Remoteness classification (ARIA)**	
Major cities	66.2%
Inner regional	29.1%
Missing/not reported	0.5%
Outside of catchment	4.2%
