# Supplementary material for: Integrity – a Driver of Integrated Care? Exploring Patient Experience in a Rapid Access, Short-Term, Outpatient Care Programme for Chronic Disease
Source: Int J Integr Care. 2026 Nov 2;26(4):6. doi: 10.5334/ijic.10199 (PMC13741939; doi:10.5334/ijic.10199)
Supplement: Supplementary file 7 [file ijic-26-4-10199-s7.pdf]

Supplementary file 7: Detailed participant characteristics

ID	Sex	Clinical Stream	Remoteness	SEIFA Decile	Age Group	First Nations
C01	Female	Respiratory	Major cities	4	65+	No
C03	Male	Respiratory	Major cities	3	65+	No
C04	Female	Cardiology	Major cities	3	65+	No
C05	Male	Cardiology	Major cities	3	65+	No
C06	Female	Respiratory	Major cities	3	65+	Yes
C07	Male	Diabetes	Major cities	7	18-29	No
C09	Female	Respiratory	Major cities	4	65+	No
C10	Female	Diabetes	Major cities	2	65+	No
C11	Male	Multistream	Major cities	3	65+	No
C12	Male	Multistream	Major cities	1	50-64	No
C13	Male	Cardiology	Inner regional	2	50-64	No
C14	Female	Multistream	Major cities	3	65+	No
C15	Female	Respiratory	Major cities	1	18-29	No
C16	Male	Cardiology	Major cities	1	65+	No
C17	Male	Respiratory	Major cities	8	50-64	No
C19	Female	Respiratory	Major cities	3	18-29	No
C20	Male	Cardiology	Major cities	6	50-64	No
C22	Female	Respiratory	Major cities	1	65+	No
C23	Female	Diabetes	Inner regional	3	50-64	No
C25	Female	Respiratory	Major cities	2	50-64	No
C27	Female	Cardiology	Major cities	2	65+	No
C28	Female	Respiratory	Major cities	8	65+	Yes
C29	Male	Multistream	Inner regional	5	65+	No
C30	Male	Multistream	Major cities	2	50-64	No
C31	Male	Respiratory	Major cities	1	65+	No
C34	Female	Respiratory	Major cities	4	65+	No
C38	Male	Diabetes	Major cities	3	30-49	No
C40	Male	Diabetes	Major cities	3	18-29	No
C42	Male	Respiratory	Inner regional	1	30-49	No
C43	Female	Diabetes	Major cities	1	50-64	No
C45	Female	Multistream	Inner regional	1	50-64	No
C46	Female	Cardiology	Major cities	3	65+	No
